# Effect of mussel‐inspired primers on resin–dentin bonding interface stability: A systematic review and meta‐analysis

**DOI:** 10.1111/eos.70011

**Published:** 2025-04-29

**Authors:** Samuel Chillavert Dias Pascoal, Maria Clara Ayres Estellita, Fábio Wildson Gurgel Costa, Juliano Sartori Mendonça

**Affiliations:** ^1^ Graduate Program in Dentistry Federal University of Ceará Fortaleza Brazil; ^2^ Department of Dentistry Federal University of Ceará Fortaleza Fortaleza Brazil; ^3^ Department of Restorative Dentistry Federal University of Ceará Fortaleza Brazil

**Keywords:** adhesion, dentin, mussel, primer

## Abstract

This paper presents the results of a systematic review and meta‐analysis on the efficacy of mussel‐inspired primers on resin‐dentin bond stability. This review was registered on the OSF platform (https://doi.org/10.17605/OSF.IO/QC7VU) and followed PRISMA guidelines. Databases included PubMed, SCOPUS, Web of Science, EMBASE, Lilacs, LIVIVO, and gray literature. Eligibility criteria included in vitro studies using human dentin, which assessed bond strength through microtensile tests after applying mussel‐inspired primers. Exclusion criteria were studies evaluating bond strength to enamel, animal dentin, and ceramics, those not using the microtensile test, or those conducting immediate evaluation only. The RoBDEMAT tool was used for risk‐of‐bias analysis. Two independent reviewers screened 5522 articles, and seven were selected. The studies showed a similar risk of bias performance, with insufficient reporting of different parameters, while not presenting significant publication bias. Meta‐analysis revealed significantly higher bond strength in specimens treated with mussel‐inspired monomers after thermocycling. Across studies, no substantial reduction in heterogeneity was observed using the leave‐one‐out method. Mussel‐inspired monomers incorporated into the dentin adhesive procedure can enhance bond strength, mainly through their catecholic groups, highlighting their potential in adhesive dentistry. However, drawbacks such as oxidative potential and effects on the degree of conversion may limit their widespread application.

## INTRODUCTION

Despite progress in the development of dental materials, effective dentin adhesion remains a challenge in adhesive dentistry [1, 2]. One problem is that adhesion is affected by morphological disparities among dental substrates. Because dentin is characterized by a higher volume of organic matrix, lower mineral content, and significantly higher water content than enamel, it is more susceptible to adhesive procedure failures [3, 4, 5]. Furthermore, the hybrid layer emerges as the critical zone for dentin adhesion, underscoring the need for strategies to mitigate this phenomenon [3, 6].

Endogenous proteases play an important role in degrading collagen fibers of the organic matrix, so protease inhibitors such as chlorhexidine (CHX) have been added to etchants, pretreatment agents, as well as incorporated into bonding systems [7, 8]. Nonetheless, there is growing interest in strategies that not only focus on inhibiting endogenous proteases but also exert biological influence on the mechanical properties of the dentin collagen [9, 10, 11, 12]. Consequently, collagen biomodifiers from natural sources, such as proanthocyanidin, epigallocatechin‐3‐gallate, and tannic acid have garnered considerable attention in studies related to dental biomaterials [12, 13]. However, drawbacks such as low substantivity, chemical instability, and substrate pigmentation limit the broad clinical application of these compounds [14, 15].

As biomaterial development increasingly seeks to replicate nature's mechanisms, the underwater environment has emerged as a promising venue for adhesion studies [16, 17, 18, 19]. Due to the ability of mussels to resist high physical turbulence, form strong ionic bonds, and adhere to low‐energy surfaces, the adhesive prowess of bivalve animals such as mussels has gained interest in the exploration of new biopolymers [16, 19, 20]. The robust adhesive capability exhibited by marine mussels in aquatic environments serves as an inspiration and a catalyst for reevaluating alternatives to enhance the stability of dentin bonding [21, 22, 23].

The unique adhesive properties of mussels are attributed to the presence of the amino acid 3,4‐dihydroxyphenylalanine, commonly known as DOPA, released by their primitive foot (byssus) [16, 17, 24]. The catecholic group in this compound possesses the capacity to form various chemical bonds with a diverse range of substrates, thereby providing adhesive properties to low‐energy regions of this molecule [25, 26, 27]. The adhesive chemical characteristics of these mussel‐inspired compounds and their recently demonstrated biocompatibility [16, 28] have spurred developments of new primers or pretreatments for adhesive dentistry to focus on the performance of these compounds.

Knowledge on the application of these compounds in adhesive dentistry is currently limited. Biomimetic agents such as mussel‐inspired adhesives have been explored in medicine for various applications in wound healing, as injectable hydrogels or scaffolds for tissue engineering, as smart detachable adhesives for hemostasis, and as biological sealants [19, 20]. However, their application in adhesive dentistry remains largely unexplored beyond in vitro studies and in vivo experiments in rat models, with no clinical trials conducted to date. The objective of this study was to perform a systematic review and meta‐analysis focused on evaluating the efficacy of mussel‐inspired primers on resin‐dentin bond stability before and after thermocycling compared with conventional adhesive systems/monomers. The study hypothesis was that the presence of mussel‐inspired molecules in primers would increase the in vitro dentin bond strength after thermocycling.

## MATERIAL AND METHODS

The present systematic review is reported in accordance with the PRISMA 2020 statement [29]. The protocol for this study was submitted to the international prospective OSF registry for general systematic reviews (https://doi.org/10.17605/OSF.IO/QC7VU, accessed on January 8, 2024). The research question formulated using the PICOT framework [30] (Table 1) was as follows: “Can mussel‐inspired primers enhance the in vitro dentin bond stability to composite resin when compared with conventional adhesive primers before and after thermocycling?”.

**TABLE 1:** Description of the PICOT protocol used to formulate the review scope question.

PICOT item	Definition
Population (P)	Sound human dentin
Intervention (I)	Mussel‐inspired primers
Comparison (C)	Commercial adhesive systems or experimental adhesives with conventional monomers
Outcome (O)	Bond strength to dentin assessed using microtensile tests
Time (T)	Aged using thermocycling

### Literature search, inclusion, and exclusion criteria

The search period spanned from July 13, 2023 to September 02, 2024. The bibliographic databases employed in this investigation included PubMed (MEDLINE), SCOPUS, Web of Science, EMBASE, LIVIVO, LILACS, and gray literature accessed via Google Scholar (Table S1). Furthermore, the reference sections of selected articles underwent manual screening to identify additional pertinent literature. Distinct search strategies were prepared for each database, adhering to prescribed formatting and employing appropriate filters. After the initial screening, files containing exported references in .ris or .nbib (PubMed files) formats were imported into mendeley reference manager desktop and endnote 20.0 software to eliminate duplicates.

#### Inclusion criteria

**FIGURE 1:**
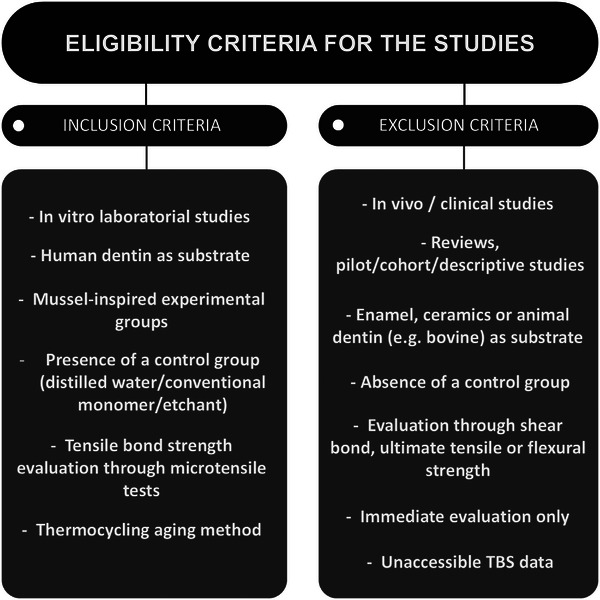
Study selection and eligibility criteria.

The inclusion criteria were in vitro laboratory studies using extracted human molars with dentin serving as the designated substrate. The intervention involved the application of primers or monomers derived from mussel‐inspired adhesives incorporated into dentin adhesive systems. Only studies featuring a control group consisting of a commercial monomer/monomeric etchant or an absence of mussel‐derived compounds in experimental formulations were considered. The outcomes of interest were the dentin bond strength, evaluated through tensile tests, conducted immediately and after a specified period of aging/storage (Figure 1). Studies in English, Portuguese, and Spanish were considered eligible, with no publication period limit.

#### Exclusion criteria

The exclusion criteria involved non‐laboratorial papers utilizing animal teeth, enamel, or ceramics as the substrates for adhesion. Studies lacking control groups or not incorporating mussel‐derived adhesive compounds as primers were also excluded. Furthermore, studies that did not perform bond strength measurements through microtensile tests or that conducted the evaluation only immediately after bonding were excluded from the analysis (Figure 1). Studies exploring the adhesiveness of compounds from underwater animals other than mussels were not considered eligible. Papers available only in repositories, papers not published in peer‐reviewed journals, and papers that had inaccessible bond strength data were removed.

### Data extraction

The screening of all imported studies post‐duplicate removal was carried out by two independent reviewers (S.C.D.P. and M.C.A.E.) on the Rayyan online platform (http://rayyan.ai). Both reviewers underwent training on the established inclusion and exclusion criteria for article selection. The decision‐making process for each reviewer was blinded, with each assigning a judgment of acceptance or rejection to all abstracts. Articles deemed potentially eligible were included in a comprehensive full‐text review. Discrepancies in decisions were resolved through consensus or consultation with a team expert (J.S.M.).

### Risk of bias assessment

The Risk of Bias (RoB) analysis was conducted by two reviewers (S.C.D.P. and M.C.A.E.) working independently. Consensus was reached after consultation with a third member (F.W.G.C.) of the research team. In order to comprehensively assess bias risk in in vitro trials, the RoBDEMAT tool [31] was used. The RoB assessment was conducted by analyzing four established domains: bias in study design and allocation, bias in sample/specimen preparation, bias in outcome assessment, and bias in data handling and reporting of results. Each item within these domains was categorized as “sufficiently reported,” “insufficiently reported,” “not reported,” or “not applicable.”

### Meta‐analysis

The parameters selected for analysis were immediate microtensile bond strength and post‐thermocycling tensile bond strength assessments. Data on sample size, mean values, and standard deviations for each study group were meticulously tabulated using the Google Spreadsheet and subsequently exported to revman 5.4 software (Cochrane.org). The meta‐analysis was computed using the inverse variance method under random effects. Heterogeneity was analyzed using I^2^ and Tau^2^ coefficients, while Egger's and Begg's tests were applied to assess the risk of publication bias.

The one‐out method was employed by systematically excluding individual study results to examine the influence of each parameter on the overall meta‐analysis. In instances where the meta‐analysis revealed a significant difference in the assessed parameters, the Cohen's d coefficient [32] was calculated to estimate the standardized mean difference between groups.

## RESULTS

**FIGURE 2:**
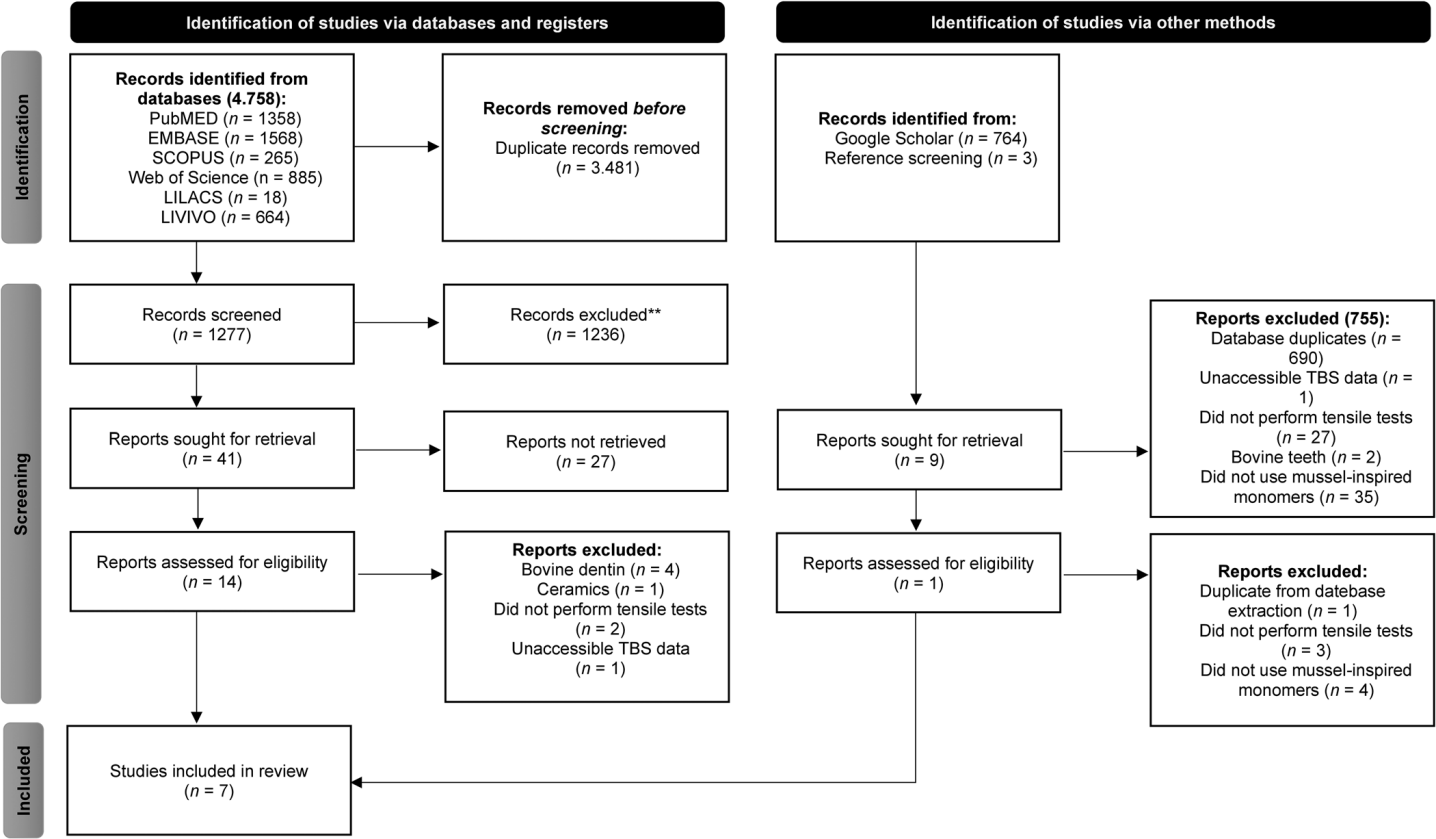
PRISMA 2020 flow diagram for systematic reviews, which included searches of databases, registers, and other sources. Out of 5124 potentially relevant papers, after screening, eligibility, and inclusion, seven remained.

A total of 5522 articles potentially relevant to the review were identified. Using both mendeley and endnote software, 3481 duplicates were successfully eliminated. The Rayyan.ai tool was then employed to evaluate 1277 articles based on their titles and abstracts, resulting in the selection of 41 for full‐text reading. To broaden the pool of potential studies for assessment, the references of each text were reviewed, leading to the inclusion of three additional studies. After a thorough examination, 14 articles were initially chosen; however, considering the predefined exclusion criteria, seven articles were ultimately selected for inclusion in the systematic review (Figure 2).

### Data extraction

The full texts were documented on the Google Spreadsheets online platform, with the extracted data comprising author names, year of publication, study objectives, experimental groups, conducted assays, application methods, materials used, microtensile results, and aging methods (Tables 2 and 3).

**TABLE 2:** Main features of the in vitro studies included regarding the materials used, primer formulation, and application instructions.

Author, year	Mussel primer composition	Control composition	pH (Mussel‐inspired groups)	Adhesive system	Composite	Application method
Fang et al. 2017 [21]	10 µL mussel adhesive protein recombinant and 40 µL of Clostridiopeptidase‐A	Distilled water	8.5	Self‐etch Gluma Comfort Bond, Kulzer	Tetric EvoCeram, Ivoclar Vivadent	Rubbing for 60 s
Li et al. 2021 [24]	Mussel‐inspired functional monomer, N‐(3,4‐dihydroxyphenethyl)methacrylamide with dimethyl sulfoxide solvent	Distilled water	Not reported	Etch‐and‐rinse Single Bond 2 3 M ESPE for 15 s	Filtek Z250, 3 M ESPE	Rubbing for 60 s
Quan‐Li et al. 2021 [26]	Mussel‐inspired functional monomer, N‐(3,4‐dihydroxyphenethyl)methacrylamide	Distilled water	2	Etch‐and‐rinse Tetric N‐Bond, Ivoclar	Tetric EvoCeram Ivoclar Vivadent	Application for 30 s
Li et al. 2021 [25]	Mussel‐inspired functional monomer, N‐(3,4‐dihydroxyphenethyl)methacrylamide with EtOH solvent	50% EtOH Distilled water	Not reported	Etch‐and‐rinse Single Bond 2 3 M ESPE for 15 s	Filtek Z250 3 M ESPE	Double application for 30 s
Xu et al. 2022 [27]	Mussel‐inspired functional monomer, N‐(3,4‐dihydroxyphenethyl)methacrylamide with phosphoric acid 1:1	37% Phosphoric acid	1.53 1.46 1.39 1.36	Etch‐and‐rinse Single Bond 2 3 M ESPE for 15 s	Filtek Z250 3 M ESPE	Application for 15 s
Hu et al. 2022 [22]	Polymerizable polymer functionalized with catechol and Lys (catechol‐Lys‐methacrylate)	Distilled water	Not reported	Etch‐and‐rinse Single Bond 2 3 M ESPE for 15 s	Filtek Z250 3 M ESPE	Rubbing for 60 s
Wu et al. 2023 [23]	Oxidized N‐2‐(3,4‐dihydroxypheny)acrylamide 5% solution	Poly‐acrylic acid, calcium phosphate, calcium chloride, water, disodium hydrogen phosphate, glycine, potassium chloride	5 9	Etch‐and‐rinse Single Bond 2 3 M ESPE for 20 s	Filtek P60 3 M ESPE	Application for 30 s

**TABLE 3:** Summary of the sample size data, groups, microtensile and thermocycling methodology, and results of selected studies.

Author, Year	*n*	Groups	Microtensile testing	Thermocycling	Results
Fang et al. 2017 [21]	15	Control: DW Positive control: 10 µL GM6001 + 40 µL Clostridiopeptidase‐A Experimental: 10 µL recombined Mfp + 40 µL Clostridiopeptidase‐A	0.8 mm^2^ specimen; 1 mm/min crosshead speed until failure	2.500 cycles, 5°C–55°C. Dwelling time of 15 s at 37°C collagenase storage for 3 weeks	After thermocycling, specimens treated with mussel‐inspired primers showed statistically significantly higher TBS values than DW
Li et al. 2021 [24]	8	Control: DW Group 1: pure DMSO; Group 2: 0.1 mmol/L DMA in DMSO; Group 3: 1.0 mmol/L DMA in DMSO; Group 4: 10 mmol/L DMA in DMSO	0.8 mm^2^ specimen; 1 mm/min crosshead speed until failure	10.000 cycles, 5°C–55°C. Dwelling time 1 min	After thermocycling, all mussel‐inspired groups presented a statistical difference to DW, with 1 mM DMA presenting the highest TBS compared with the control group
Quan‐Li et al. 2021 [26]	25	Control: DW Experimental 1: 2 mg/mL dopamine methacrylamide (DMA)	0.9 mm^2^ specimen; 0.5 mm/min crosshead speed until failure	2.500 cycles, 5°C–55°C. Dwelling time 15 s	After thermocycling, specimens treated with DMA primer showed statistically significantly higher TBS values than controls
Li et al. 2021 [25]	10	Control: 50% EtOH Experimental 1: 1 mmol/L DMA‐EtOH (1 mM DMA); Experimental 2: 5 mmol/L DMA‐EtOH (5 mM DMA); Experimental 3: 10 mmol/L DMA‐EtOH (10 mM DMA)	0.9 × 0.9 × 8 mm specimen; 1 mm/min crosshead speed until failure	10.000 cycles, 5°C–55°C. Dwelling time of 15 s transfer 7 s	After thermocycling, specimens treated with mussel‐inspired primers showed statistically significantly higher TBS values than controls
Xu et al. 2022 [27]	5	Control: phosphoric acid Solvent control: DMSO + phosphoric acid (1:1) Experimental 1: 1 mmol/L DMA in DMSO; Experimental 2: 3 mmol/L DMA in DMSO; Experimental 3: 5 mmol/L DMA in DMSO	1 × 1 × 6 mm specimen; 1 mm/min crosshead speed until failure	20.000 cycles, 10°C–55°C. Dwelling time 60 s	After thermocycling, specimens treated with 5 mM DMA‐DMSO showed statistically significantly higher TBS values than controls
Hu et al. 2022 [22]	32	Control: Distilled water Experimental 1: 1 mg/mL CLM Experimental 2: 5 mg/mL CLM Experimental 3: 10 mg/mL CLM	1 × 1 × 10 mm specimen; 1 mm/min crosshead speed until failure	10.000 cycles, 5°C–55°C. Dwelling time 60 s	At immediate level and post‐thermocycling, specimens treated with 5 mg/mL CLM showed statistically significantly higher TBS values than controls
Wu et al. 2023 [23]	20	Control: conventional monomer Experimental: 5% OX‐DAA	1 × 1 × 8 mm specimen; 1 mm/min crosshead speed until failure	2.500 cycles, 5°C–55°C. Dwelling time 15 s at 37°C collagenase storage for 3 weeks	After thermocycling, specimens treated with 5% OX‐DAA showed statistically significantly higher TBS values than the DW group

Abbreviations: CLM, catechol‐lysine‐methacrylate; DMA, dopamine methacrylamide; DMSO, dimethyl sulfoxide; DW, distilled water; EtOH, ethanol; GM6001, synthetic broad spectrum metalloproteinase inhibitor; Mfp, mussel‐foot protein; OX‐DAA, oxidized N‐2‐(3,4‐dihydroxypheny)acrylamide; TBS, tensile bond strength.

### Systematic review

All the selected studies conducted bond strength evaluations using microtensile tests. The formulations of the experimental primers/pretreatments varied in terms of the solvent and/or the mussel‐derived compound used, including amino acids such as DOPA and whole mussel‐foot proteins (Mfp). The control groups ranged from distilled water used as a treatment control for commercial adhesives to nonbioinspired conventional monomers/etchants. The methodologies for thermocycling varied across studies, ranging from 2500 to 20,000 cycles. One study included an experimental adhesive with conventional monomer as the control in their formulations [23], while the remaining studies used distilled water as a control. Only one study incorporated a mussel functional monomer in the etching agent, while the others used it as dentin primers [27] (Table 2).

### Risk of bias analysis

The risk of bias and the analyzed domains are presented in Table 4. All selected studies defined and sufficiently reported a control group. However, regarding the domains of specimen randomization, sample size calculation, and blinding of test operators, none of the papers were sufficiently reported. Additionally, concerning the standardization of results and appropriate testing, all manuscripts were reported as inadequately reported. Records classified as insufficiently reported in the presented results may be attributed to either not conducting a failure mode analysis or omitting the TBS values for specific groups.

**TABLE 4:** RoBDEMAT bias assessment table for each domain classified as “sufficiently reported,” “insufficiently reported,” and “not reported” [31].

	D1: Bias in planning and allocation			D2: Bias in specimen preparation		D3: Bias in outcome assessment		D4: Bias in data treatment and reporting	
Study	Control group	Sample size calculation	Correct randomization of samples	Identical experimental conditions	Standardization of samples and materials	Adequate and standardized testing procedures/outcomes	Blinding of the testing operator	Appropriate statistical analysis	Correct reporting of outcomes
Fang et al. 2017 [21]	Sufficiently reported	Not reported	Not reported	Sufficiently reported	Sufficiently reported	Insufficiently reported	Not reported	Sufficiently reported	Insufficiently reported
Li et al. 2021 [24]	Sufficiently reported	Not reported	Not reported	Sufficiently reported	Sufficiently reported	Insufficiently reported	Not reported	Sufficiently reported	Sufficiently reported
Quan‐Li et al. 2021 [26]	Sufficiently reported	Not reported	Not reported	Sufficiently reported	Sufficiently reported	Insufficiently reported	Not reported	Sufficiently reported	Insufficiently reported
Li et al. 2021 [25]	Sufficiently reported	Not reported	Not reported	Sufficiently reported	Sufficiently reported	Insufficiently reported	Not reported	Sufficiently reported	Sufficiently reported
Xu et al. 2022 [27]	Sufficiently reported	Not reported	Not reported	Insufficiently reported	Sufficiently reported	Insufficiently reported	Not reported	Sufficiently reported	Sufficiently reported
Hu et al. 2022 [22]	Sufficiently reported	Not reported	Not reported	Sufficiently reported	Sufficiently reported	Insufficiently reported	Not reported	Sufficiently reported	Sufficiently reported
Wu et al. 2023 [23]	Sufficiently reported	Not reported	Not reported	Insufficiently reported	Insufficiently reported	Insufficiently reported	Not reported	Not reported	Sufficiently reported

### Quantitative results

**FIGURE 3:**
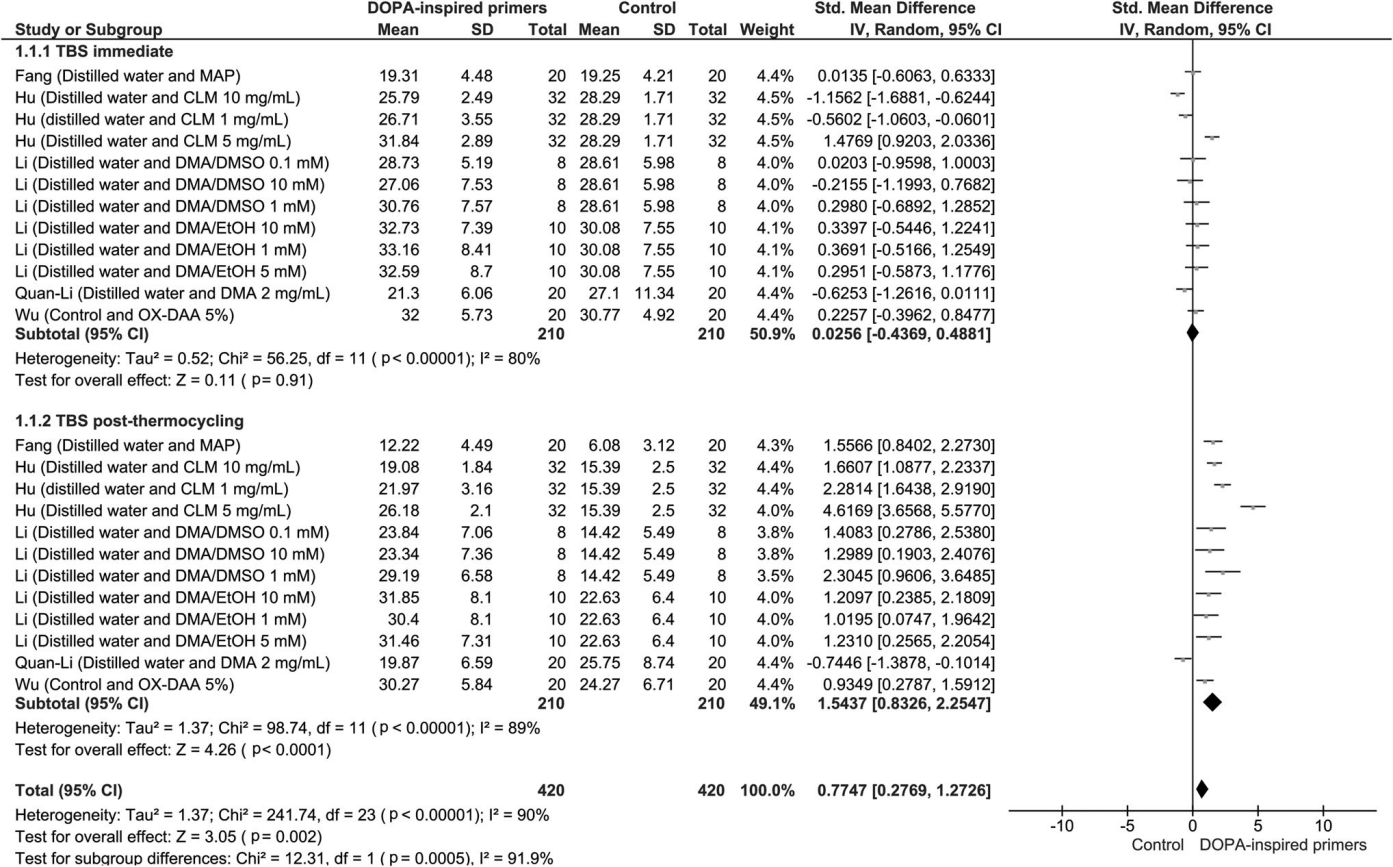
Evaluation by meta‐analysis of the dentin/resin bonding stability of the DOPA‐inspired primers/pretreatments in the adhesive process.

A meta‐analysis was conducted for two time points, immediately after bonding and after thermocycling. Among the selected papers, the study by Xu et al. [27] was excluded because it was the sole article that incorporated mussel‐inspired pretreatment in the etching agent, preventing the inclusion of this dataset in the analysis (Figure 3). Importantly, it was not possible to perform multiple subgroup comparison analyses such as adhesive strategies or primer formulations, since only Fang et al. [21] performed the adhesive procedure in self‐etch mode, while only Hu et al. [22] conducted a methacrylate mussel‐incorporation.

Regarding the analysis of bond strength values obtained immediately after bonding, the difference in mean TBS values between the groups was not statistically significant (mean difference = 0.03 MPa, 95% CI = –0.44 to 0.49). For the subgroup analysis of bond strength post‐thermocycling, a statistically significantly higher (*p* < 0.0001) bond strength was observed for groups treated with mussel‐derived monomers, with an average difference of 1.54 MPa (95% CI = 0.83–2.25). This difference can be characterized by a large effect size (Cohen's d = 2.15). Significant heterogeneity between studies was observed (*p* < 0.00001, I^2^ = 89%, Tau^2^ = 1.37). The exclusion of individual study findings through a one‐at‐a‐time analysis did not result in a substantial change in the estimated effect or the heterogeneity among the studies.

**FIGURE 4:**
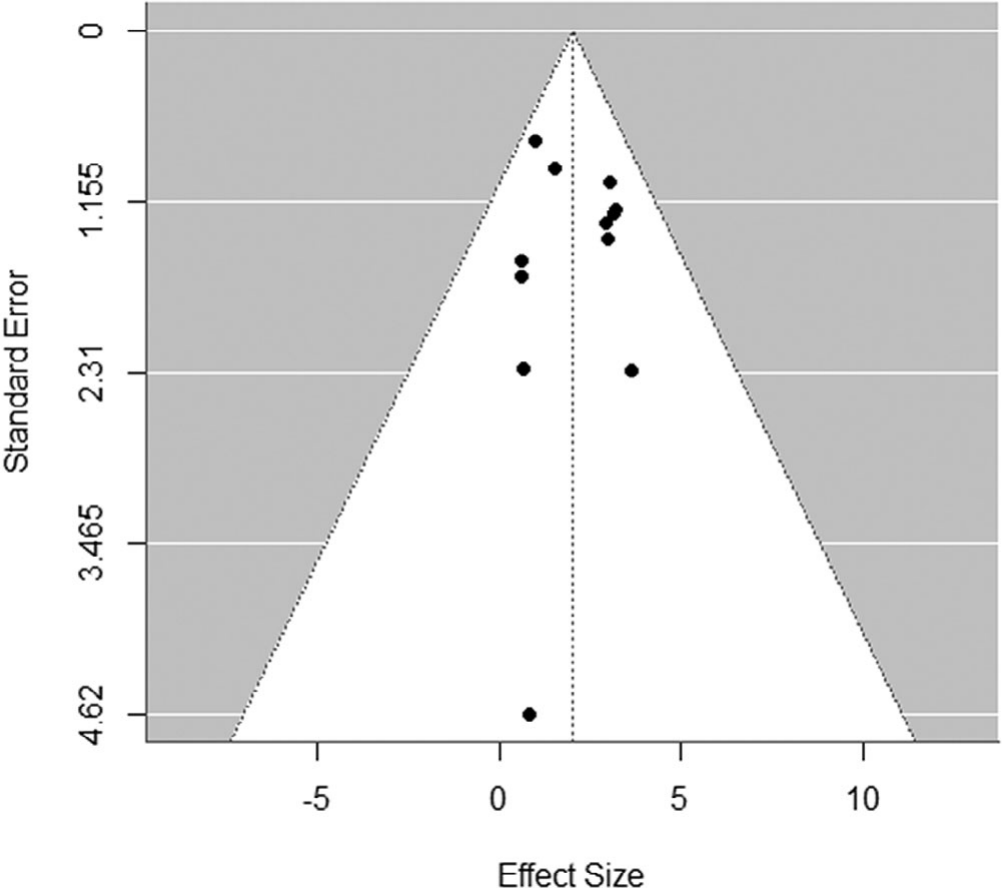
The low risk of publication bias (Egger's test, *p* = 0.74; Begg's test, *p* = 0.49).

The results of the publication bias assessment indicated that there was no statistically significant evidence of publication bias in the studies included in this meta‐analysis. The Egger test, which assessed asymmetry in the funnel plot (Figure 4), resulted in a *p*‐value of 0.74, while the Begg's test, which examines the correlation between the standardized treatment effect and the variance of the treatment effect, resulted in a *p*‐value of 0.49. Both *p*‐values were above the conventional threshold for statistical significance (*α* = 0.05), not indicating a publication bias.

## DISCUSSION

This systematic review and meta‐analysis organized data from in vitro studies on the bond strength of composite resin to human dentin after thermocycling, employing experimental mussel‐inspired monomers. The findings revealed that dentin treated with these mussel‐inspired adhesive monomers generally exhibited higher bond strength after thermocycling than the control specimens, in which bonding involved experimental primers with conventional monomers or commercial adhesive materials. After thermocycling, the difference amounted to an average of 1.54 MPa (95% CI = 0.83–2.25) higher bond strength in specimens treated with mussel‐inspired adhesives than in control specimens.

The ester‐type bonds within commercial primers are particularly susceptible to water molecules, which hastens their biodegradation [25, 26]. To counteract this phenomenon, the integration of monomers inspired by the underwater adhesiveness of mussels, whether in primers, pretreatments, or even in conditioning agents such as phosphoric acid, emerges as an alternative for enhanced chemical action [22, 27, 28]. A key collagen biomodification mechanism involves the strong adsorption of the DOPA‐catechol group to dentin, which engages in divalent hydrogen bonding along the edges of the hydroxyapatite crystal [26]. Beyond this strategy, there is evidence of the utilization of other mussel‐derived amino acids, such as catecholic lysines, oxidized versions of DOPA, and even the application of whole mussel‐foot proteins [21, 22, 23]. Additionally, there is potential for the use of functionalized monomers in primers already in polymer form with biological dentin action, as reported in some studies [28].

When DOPA is incorporated into the methacrylamide monomer, a significantly higher dentin bond strength is observed [6, 25, 27]. Although this effect may not be sufficient to exhibit statistical differences relative to control groups immediately after bonding, there are reports of sustained strength after aging through simulated thermocycling, representing periods ranging from 3 months to 2 years. At a molecular level, outcomes of molecular docking studies suggest the presence of covalent bonds, hydrogen bonds, and van der Waals attractive forces as mussel‐inspired monomers interact with collagen fibrils [22, 27]. Methacrylamide monomers can form hydrogen bonds with water and act as both donors and acceptors of hydrogen bonds, O–H and NH dipoles, respectively [32, 33]. Other derivatives of DOPA subjected to oxidation, such as dopamine [27], can be incorporated into methacrylamide, where the terminal structure of the carbon–carbon double bond (CH_2_ = CH–) in the monomer can copolymerize with the adhesive monomer to form a chemical linkage [16, 25]. It is noteworthy that either natural or synthetic adhesive proteins containing oxidized catechols can be detrimental to their adhesive ability because the formed o‐quinone links are non‐adhesive [23]. However, oxidized mussel monomers containing catechin and imine groups, similar to the functional groups of dopamine and lysine in mussel adhesion protein molecules that interact with the collagen matrix, may promote the maintenance of dentin bond strength even after thermocycling [22, 23, 27].

Even though the methacrylate group is inherently more hydrophilic than methacrylamides, the potential to functionalize mussel‐derived molecules into methacrylates holds the promise of achieving superior dentin adhesion [22, 27, 34]. In contrast to other pretreatment strategies, this functionalization aims to eliminate the need for additional steps and to maintain a user‐friendly application [27]. Due to the lower electronegativity of oxygen atoms in the methacrylate group compared with nitrogen atoms in amides, coupled with the biological action present in mussel‐inspired molecules, an increase in dentin bond strength was also achieved [16, 26]. Moreover, the addition of ions such as Fe^+^ in polymers doped with mussel‐derived monomers could enhance the bond strength in dentin contaminated by saliva and mucin [28]. Possibly, the catechol groups in the polymeric chains can bind to dentin surfaces through hydrogen bonding or chelation with calcium in hydroxyapatite minerals, where Fe facilitates the formation of complex catechol crosslinks within its polymeric chains in the presence of water [16, 22, 28].

Considering that most microtensile tests in the selected studies employed etch‐and‐rinse adhesive systems, the addition of an extra step involving the application of pretreatments/primers tends to heighten the complexity of the procedure and extend the clinical chair time [27, 35].

Thus, the possibility of incorporating mussel‐inspired functional monomers into etching agents, such as phosphoric acid, has also been suggested [27], not limiting the use of DOPA and its derivatives to primers/pretreatments. Because its pH ranges between 1 and 2, the experimental etchant used may be classified as a mild‐to‐strong etching strategy [27]. During etching, the concurrent application of functionalized primers prevents the enzymolysis of exposed collagen fibrils, as an acidic environment activates matrix metalloproteinases and cysteine cathepsins entrapped in mineralized dentin [22, 25, 27]. Despite showing a significant reduction in bond strength after aging, more acidic concentrations of the monomer exhibited the lowest rates of interface biodegradation [27].

The role of pH in the effectiveness of these bioinspired monomers is underscored because the acidity or alkalinity of the environment can alter the pharmacokinetics of these dentinal “cocktails” [23, 27]. In the selected studies, the pH of the primers varied significantly from 1.36 to 9, indicating a range of chemical mechanisms of action for these compounds. In certain formulations, mussel‐inspired monomers under acidic conditions retain nonoxidized phenolic hydroxyl groups, promoting crosslinking with collagen through amino acids containing side chains –COOH and –NH_2_, while –OH primarily interacts through hydrogen bonds with the same amine and carboxyl groups [23]. Conversely, under alkaline conditions, the phenolic hydroxyl group in the DOPA molecule is readily oxidized to quinone, enabling the crosslinking of the oxidized monomer with collagen fibrils through amino acids containing side chains –NH_2_, and –C = O interactions occur primarily through the Schiff base reaction with amines [23].

Not only can hydrolytic degradation be a detrimental factor to the integrity of the hybrid layer, but the collagenolytic activity of endogenous proteases, such as matrix metalloproteinases (MMPs) and cathepsins (CTPs), is known to significantly reduce the bond strength of composite resin restorations [3, 6, 18]. Given that amino acids derived from mussels demonstrate a proven affinity for metallic ions, it is plausible that catalytic ions, such as Zn^2+^ and Ca^2+^, in MMPs may be chelated by functional monomers [26]. This interaction can deactivate endogenous proteases by three‐dimensionally modifying their molecules, resulting in a reduction of activation sites at the adhesive interface [6, 20, 25]. It has also been proposed that monomers inspired by mussels can form covalent cross‐links with collagen fibers, preserving the stability of the collagen triple helix [21, 23, 25]. This process is believed to hinder collagenase from linking to the enzyme binding site, effectively preventing collagen fibril degradation [21, 22, 28].

It is also important to report on the adhesive strategies employed in the included studies, as the vast majority favor the etch‐and‐rinse system, in contrast to the self‐etching system used in only two of seven studies. Simplified dental adhesive systems, such as universal adhesives, tend to be more susceptible to hydrolytic/proteolytic degradation given the hydrophilic nature of their polymeric chains [32, 36]. Particularly for universal adhesives in the self‐etching mode, their ability to maintain bond strength after storage or aging may decrease significantly [34, 37, 38]. This information highlights a relevant gap in the field of the adhesive efficacy of mussel‐inspired monomers, indicating the need for evaluation in terms of different adhesive strategies or substrates.

It is important to note certain limitations regarding the use of bio‐inspired primers for mussel adhesion. At higher concentrations, DOPA molecules incorporated into a methacrylate monomer can significantly reduce the degree of conversion of an experimental adhesive [6, 22]. Moreover, DOPA can be easily oxidized in the presence of quinone [25]. Although this oxidation sometimes leads to a favorable increase in TBS [21, 23], the potential for primer‐induced discoloration at the adhesive interface may prove undesirable, thus discouraging use in esthetic restorations [12, 13, 30].

Regarding the literature review, it is imperative to acknowledge the potential presence of biases that may affect the overall quality and reliability of the results. Although the meta‐analysis shows a significant increase in bond stability after thermocycling, the RoB analysis shows bias in several key parameters related to the experimental design of the studies, which may reduce the reliability of the data. Moreover, the high heterogeneity observed in the quantitative assessment between the mussel‐inspired groups is consistent with the risk of bias assessment and may reduce confidence in the results.

Considering that investigating underwater adhesion mechanisms is a relatively new field, the lack of positive controls can create methodological gaps, even if all studies have presented a negative control group. Efficacy benchmarks are essential points for assessing whether mussel‐derived monomers are at least as effective as existing approaches or surpass the standard treatment, thereby validating the experimental design. Despite one of the eligibility criteria for this review being performance assessment through microtensile tests, the omission of results regarding failure modes can compromise the interpretation of the results on bond strength in adhesive interfaces, thus hindering the development of effective clinical interventions.

The use of these mussel‐inspired monomers is promising in the field of adhesion. Given their potential for wet adhesion on diverse substrates, evaluating their adhesiveness on ceramic surfaces or other dental tissues is desirable. As studies move toward a more user‐friendly approach, it would be interesting to observe the effectiveness of these amino acids/proteins incorporated into different as well as simpler adhesive systems, such as a single‐step self‐etching or universal adhesives, particularly considering their effects at in vivo and clinical levels.

Considering its wet adhesion potential, the application of mussel‐inspired compounds in adhesion can effectively enhance the resin‐dentin bonding interface after thermocycling when used as primers only or being incorporated into conditioning agents such as phosphoric acid. However, it is important to consider the impact of their high oxidative potential and their impact on the degree of conversion at certain concentrations.

## AUTHOR CONTRIBUTIONS

**Conceptualization**: Samuel Chillavert Dias Pascoal, Fábio Wildson Gurgel Costa, Juliano Sartori Mendonça; **Investigation**: Samuel Chillavert Dias Pascoal, Maria Clara Ayres Estellita; **Data curation**: Samuel Chillavert Dias Pascoal, Maria Clara Ayres Estellita; **Formal analysis**: Samuel Chillavert Dias Pascoal, Maria Clara Ayres Estellita; **Investigation**: Samuel Chillavert Dias Pascoal, Maria Clara Ayres Estellita; **Methodology**: Samuel Chillavert Dias Pascoal, Maria Clara Ayres Estellita; **Visualization**: Samuel Chillavert Dias Pascoal, Maria Clara Ayres Estellita, Juliano Sartori Mendonça; **Writing—original draft**: Samuel Chillavert Dias Pascoal; **Writing—review & editing**: Fábio Wildson Gurgel Costa; **Supervision**: Fábio Wildson Gurgel Costa, Juliano Sartori Mendonça; **Project administration**: Juliano Sartori Mendonça.

## CONFLICT OF INTEREST STATEMENT

The authors declare no conflicts of interest.

## Supporting information



Supporting Information
