# Supplementary material for: Effect of mussel‐inspired primers on resin–dentin bonding interface stability: A systematic review and meta‐analysis
Source: Eur J Oral Sci. 2025 Apr 29;133(3):e70011. doi: 10.1111/eos.70011 (PMC12092816; doi:10.1111/eos.70011)
Supplement: Supplementary file 1 — Supporting Information [file EOS-133-e70011-s001.pdf]

SUPPORTING INFORMATION

Effect of mussel-inspired primers on resin-dentin bonding interface stability: a systematic review and meta-analysis

PASCOAL SCD, ESTELLITA MCA, COSTA FWG, MENDONÇA JS

Federal University of Ceará, Fortaleza, Brazil

Table S1. Search strategy used in each database to retrieve full-text papers. Search strategy used in the databases for the systematic review, including search terms, filters used and boolean operators applied in PubMed, SCOPUS, Web of Science, EMBASE, Lilacs, LIVIVO, and Google Scholar.

Database	Filter	Search strategy
PubMed (MEDLINE)	No filters used	("Dihydroxyphenylalanine"[Mesh] OR "adhesive protein, mussel" [Supplementary Concept] OR "3,4 Dihydroxyphenylalanine"[Title/Abstract] OR "L-3,4 Dihydroxyphenylalanine"[Title/Abstract] OR "L-3,4 Dihydroxyphenylalanine"[Title/Abstract] OR "L-Dopa"[Title/Abstract] OR "DOPA"[Title/Abstract] OR "adhesive protein (mussel)"[Title/Abstract] OR "mussel adhesive protein"[Title/Abstract] OR "mussel"[Title/Abstract] OR "mussels"[Title/Abstract]) AND ("Dentin-Bonding Agents"[Mesh] OR "Agents, Dentin-Bonding"[Title/Abstract] OR "Dental Bonding"[Mesh] OR "Adhesives"[Mesh] OR "Adhesive"[Title/Abstract] OR "Adhesives/therapeutic use"[Mesh] OR "monomer"[Title/Abstract])
EMBASE	No filters used	#1 'DOPA'/exp OR 'DOPA' OR '3 (3, 4 dihydroxyphenyl) d alanine' OR '3 (3, 4 dihydroxyphenyl) dl alanine' OR '3 (3, 4 dihydroxyphenyl) alanine' OR '3, 4 dihydroxy dl phenylalanine' OR '3, 4 dihydroxy phenyl alanine' OR '3, 4 dihydroxyphenylalanine' OR 'd, l dopa' OR 'dextrolevo dopa' OR 'dihydroxyphenylalanine' OR 'dl 3 (3, 4 dihydroxyphenyl) alanine' OR 'dl 3, 4 dihydroxyphenylalanine' OR 'dl dopa' OR 'dopa reaction' OR 'adhesive protein mussel'/exp OR 'adhesive protein mussel' OR 'levodopa'/exp OR '3 (3, 4 dihydroxyphenyl) levo alanine' OR '3, 4 dihydroxyphenyl l alanine' OR 'l 3, 4 dihydroxyphenylalanine' OR 'l dihydroxyphenylalanine' OR 'l dopa' OR 'levo 3 (3, 4 dihydroxyphenyl) l alanine' OR 'levo 3, 4 dihydroxyphenylalanine' OR 'levo beta (3, 4 dihydroxyphenyl) alanine' OR 'levo dopa' OR 'levodopa' OR 'mussel'/exp OR 'mussels' OR 'mussel' OR 'mussel foot protein'/exp OR 'mussel foot protein' OR 'mussel extract'/exp OR 'mussel extract'<br><br>#2 'dentin bonding agent'/exp OR 'dentin bonding agents' OR 'dentin-bonding agents' OR 'dentin bonding agent' OR 'dental bonding'/exp OR 'dental bonding' OR 'adhesive agent'/exp OR 'adhesive' OR 'adhesives' OR 'adhesive agent' OR 'monomer'/exp OR 'monomery' OR 'monomer'<br><br>#3 'DOPA'/exp OR 'DOPA' OR '3 (3, 4 dihydroxyphenyl) d alanine' OR '3 (3, 4 dihydroxyphenyl) dl alanine' OR '3 (3, 4 dihydroxyphenyl) alanine' OR '3, 4 dihydroxy dl phenylalanine' OR '3, 4 dihydroxy phenyl alanine' OR '3, 4

		dihydroxyphenylalanine' OR 'd, l dopa' OR 'dextrolevo dopa' OR 'dihydroxyphenylalanine' OR 'dl 3 (3, 4 dihydroxyphenyl) alanine' OR 'dl 3, 4 dihydroxyphenylalanine' OR 'dl dopa' OR 'dopa reaction' OR 'adhesive protein mussel'/exp OR 'adhesive protein mussel' OR 'levodopa'/exp OR '3 (3, 4 dihydroxyphenyl) levo alanine' OR '3, 4 dihydroxyphenyl l alanine' OR 'l 3, 4 dihydroxyphenylalanine' OR 'l dihydroxyphenylalanine' OR 'l dopa' OR 'levo 3 (3, 4 dihydroxyphenyl) l alanine' OR 'levo 3, 4 dihydroxyphenylalanine' OR 'levo beta (3, 4 dihydroxyphenyl) alanine' OR 'levo dopa' OR 'levodopa' OR 'mussel'/exp OR 'mussels' OR 'mussel' OR 'mussel foot protein'/exp OR 'mussel foot protein' OR 'mussel extract'/exp OR 'mussel extract' AND ('dentin bonding agent'/exp OR 'dentin bonding agents' OR 'dentin-bonding agents' OR 'dentin bonding agent' OR 'dental bonding'/exp OR 'dental bonding' OR 'adhesive agent'/exp OR 'adhesive' OR 'adhesives' OR 'adhesive agent' OR 'monomer'/exp OR 'monomery' OR 'monomer')
SCOPUS	No filters used	"#1 “DOPA” OR “L-3,4 Dihydroxyphenylalanine” OR ""L-3,4 Dihydroxyphenylalanine"" OR ""L-Dopa"" OR ""3 (3, 4 dihydroxyphenyl) d alanine"" OR “3 (3, 4 dihydroxyphenyl) dl alanine” OR “3 (3, 4 dihydroxyphenyl) alanine” OR “3, 4 dihydroxy dl phenylalanine” OR “3, 4 dihydroxy phenyl alanine” OR “3, 4 dihydroxyphenylalanine” OR “d, l dopa” OR “dextrolevo dopa” OR “dihydroxyphenylalanine” OR “dl 3 (3, 4 dihydroxyphenyl) alanine” OR “dl 3, 4 dihydroxyphenylalanine” OR “dl dopa” OR “dopa reaction” OR “adhesive protein mussel”/exp OR “adhesive protein mussel” OR “levodopa”/exp OR “3 (3, 4 dihydroxyphenyl) levo alanine” OR “3, 4 dihydroxyphenyl l alanine” OR “l 3, 4 dihydroxyphenylalanine” OR “l dihydroxyphenylalanine” OR “l dopa” OR “levo 3 (3, 4 dihydroxyphenyl) l alanine” OR “levo 3, 4 dihydroxyphenylalanine” OR “levo beta (3, 4 dihydroxyphenyl) alanine” OR “levo dopa” OR “levodopa” OR “mussel” OR “mussels” OR “mussel foot protein” OR “mussel extract”<br><br>#2 “dentin bonding agent” OR “dentin bonding agents” OR “dentin-bonding agents” OR “dental bonding” OR “adhesive” OR “adhesives” OR “adhesive agent” OR “monomery” OR “monomer”<br><br>#3 TITLE-ABS-KEY (“DOPA” OR “L-3,4 Dihydroxyphenylalanine” OR ""L-3,4 Dihydroxyphenylalanine"" OR ""L-Dopa"" OR ""3 (3, 4 dihydroxyphenyl) d alanine"" OR “3 (3, 4 dihydroxyphenyl) dl alanine” OR “3 (3, 4 dihydroxyphenyl) alanine” OR “3, 4 dihydroxy dl phenylalanine” OR “3, 4 dihydroxy phenyl alanine” OR “3, 4 dihydroxyphenylalanine” OR “d, l dopa” OR “dextrolevo dopa” OR “dihydroxyphenylalanine” OR “dl 3 (3, 4 dihydroxyphenyl) alanine” OR “dl 3, 4 dihydroxyphenylalanine” OR “dl dopa” OR “dopa reaction” OR “adhesive protein mussel”/exp OR “adhesive protein mussel” OR “levodopa”/exp OR “3 (3, 4 dihydroxyphenyl) levo alanine” OR “3, 4 dihydroxyphenyl l alanine” OR “l 3, 4 dihydroxyphenylalanine” OR “l dihydroxyphenylalanine” OR “l dopa” OR “levo 3

		(3, 4 dihydroxyphenyl) l alanine” OR “levo 3, 4 dihydroxyphenylalanine” OR “levo beta (3, 4 dihydroxyphenyl) alanine” OR “levo dopa” OR “levodopa” OR “mussel” OR “mussels” OR “mussel foot protein” OR “mussel extract”) AND TITLE-ABS-KEY (“dentin bonding agent” OR “dentin bonding agents” OR “dentin-bonding agents” OR “dental bonding” OR “adhesive” OR “adhesives” OR “adhesive agent” OR “monomery” OR “monomer”)"
Web of Science	No filters used	(“DOPA” OR “L-3,4 Dihydroxyphenylalanine” OR "L-Dopa" OR "3 (3, 4 dihydroxyphenyl)” OR “dl alanine” OR “3, 4 dihydroxy phenyl alanine” OR “3, 4 dihydroxyphenylalanine” OR “d, l dopa” OR “dihydroxyphenylalanine” OR “dl 3 (3, 4 dihydroxyphenyl) alanine” OR “dl 3, 4 dihydroxyphenylalanine” OR “dl dopa” OR “adhesive protein mussel” OR “adhesive protein mussel” OR “levodopa” OR “3, 4 dihydroxyphenyl l alanine” OR “l 3, 4 dihydroxyphenylalanine” OR “l dihydroxyphenylalanine” OR “l dopa” OR “levo 3 (3, 4 dihydroxyphenyl) l alanine” OR “levo 3, 4 dihydroxyphenylalanine” OR “levo dopa” OR “levodopa”) and (“dentin bonding agent” OR “dentin bonding agents” OR “dentin-bonding agents” OR “dental bonding” OR “adhesive” OR “adhesives” OR “adhesive agent”)
LIVIVO	Medline entries excluded	(“DOPA” OR “L-3,4 Dihydroxyphenylalanine” OR "L-3,4 Dihydroxyphenylalanine" OR "L-Dopa" OR "3 (3, 4 dihydroxyphenyl) d alanine" OR “3 (3, 4 dihydroxyphenyl) dl alanine” OR “3 (3, 4 dihydroxyphenyl) alanine” OR “3, 4 dihydroxy dl phenylalanine” OR “3, 4 dihydroxy phenyl alanine” OR “3, 4 dihydroxyphenylalanine” OR “d, l dopa” OR “dextrolevo dopa” OR “dihydroxyphenylalanine” OR “dl 3 (3, 4 dihydroxyphenyl) alanine” OR “dl 3, 4 dihydroxyphenylalanine” OR “dl dopa” OR “dopa reaction” OR “adhesive protein mussel” OR “adhesive protein mussel” OR “levodopa” OR “3 (3, 4 dihydroxyphenyl) levo alanine” OR “3, 4 dihydroxyphenyl l alanine” OR “l 3, 4 dihydroxyphenylalanine” OR “l dihydroxyphenylalanine” OR “l dopa” OR “levo 3 (3, 4 dihydroxyphenyl) l alanine” OR “levo 3, 4 dihydroxyphenylalanine” OR “levo beta (3, 4 dihydroxyphenyl) alanine” OR “levo dopa” OR “levodopa” OR “mussel” OR “mussels” OR “mussel foot protein” OR “mussel extract”) AND (“dentin bonding agent” OR “dentin bonding agents” OR “dentin-bonding agents” OR “dental bonding” OR “adhesive” OR “adhesives” OR “adhesive agent” OR “monomery” OR “monomer”)
LILACS	Medline entries excluded	("Dopa" OR "L-3,4 Dihydroxyphenylalanine" OR "Di-Hidroxifenilalanina" OR "Levodopa" OR "L-Dopa" OR "3 (3, 4 dihydroxyphenyl)" OR "dl alanine" OR "3, 4 dihydroxy phenyl alanine" OR "3, 4 dihydroxyphenylalanine" OR "3,4-Di-Hidroxifenilalanina" OR "d, l dopa" OR "dihydroxyphenylalanine" OR "dl 3 (3, 4 dihydroxyphenyl) alanine" OR "dl 3, 4 dihydroxyphenylalanine" OR "dl dopa" OR "mussel" OR "mexilhões" OR "bivalvia" OR "moluscos bivalves" OR "adhesive protein mussel" OR "adhesive protein mussel" OR "levodopa" OR "3, 4 dihydroxyphenyl l alanine" OR "l 3, 4 dihydroxyphenylalanine" OR "l dihydroxyphenylalanine" OR "l dopa" OR "levo 3 (3, 4 dihydroxyphenyl) l alanine" OR "levo 3, 4 dihydroxyphenylalanine" OR "levo dopa” OR “levodopa") AND

		("dentin bonding agent" OR "Agente de Adesão Dentinária" OR "Adesivo Dentinário" OR "dentin bonding agents" OR "dentin-bonding agents" OR "dental bonding" OR "colagem dentária" OR "Aderência dentária" OR "Aderência dental" OR "adhesive" OR "adesivo" OR "adesivos dentários" OR "adhesives" OR "adhesive agent")
Google Scholar	Citations and patents excluded	("Mussel") AND ("Adhesive") AND ("Dentin") AND ("Bonding")
